# Seagrass Ecosystem Services and Their Variability across Genera and Geographical Regions

**DOI:** 10.1371/journal.pone.0163091

**Published:** 2016-10-12

**Authors:** Lina Mtwana Nordlund, Evamaria W. Koch, Edward B. Barbier, Joel C. Creed

**Affiliations:** 1 Department of Ecology, Environment and Plant Sciences, Stockholm University, SE-106 91, Stockholm, Sweden; 2 Horn Point Laboratory, University of Maryland Center for Environmental Science, Cambridge, MD, 21613, United States of America; 3 Department of Economics and Finance, University of Wyoming, 1000 E. University Ave., Laramie, WY, 82071, United States of America; 4 Laboratório de Ecologia Marinha Bêntica, Departamento de Ecologia, Instituto de Biologia Roberto Alcântara Gomes, Universidade do Estado do Rio de Janeiro – UERJ, PHLC Sala 220, Rua São Francisco Xavier 524, CEP 20559-900, Rio de Janeiro, RJ, Brazil; USDA-ARS Fort Keogh Livestock and Range Research Laboratory, UNITED STATES

## Abstract

Threats to and loss of seagrass ecosystems globally, impact not only natural resources but also the lives of people who directly or indirectly depend on these systems. Seagrass ecosystems play a multi-functional role in human well-being, e.g. food through fisheries, control of erosion and protection against floods. Quantifying these services reveals their contributions to human well-being and helps justify seagrass conservation. There has been no comprehensive assessment as to whether seagrass ecosystem services are perceived to vary over the globe or amongst genera. Our study compiles the most complete list of ecosystem services provided by seagrasses so far, including bioregional- and genus-specific information from expert opinion and published studies. Several seagrass ecosystem services vary considerably in their (known) provision across genera and over the globe. Seagrasses genera are clearly not all equal with regard to the ecosystem services they provide. As seagrass genera are not evenly distributed over all bioregions, the presence of an ecosystem service sometimes depends on the genera present. Larger sized seagrass genera (e.g. *Posidonia*, *Enhalus*) are perceived to provide more substantial and a wider variety of ecosystem services than smaller species (e.g. *Halophila*, *Lepilaena*). Nevertheless, smaller species provide important services. Our findings point out data gaps, provide new insight for more efficient management and recommend caution in economic valuation of seagrass services worldwide.

## Introduction

**Fig 1:**
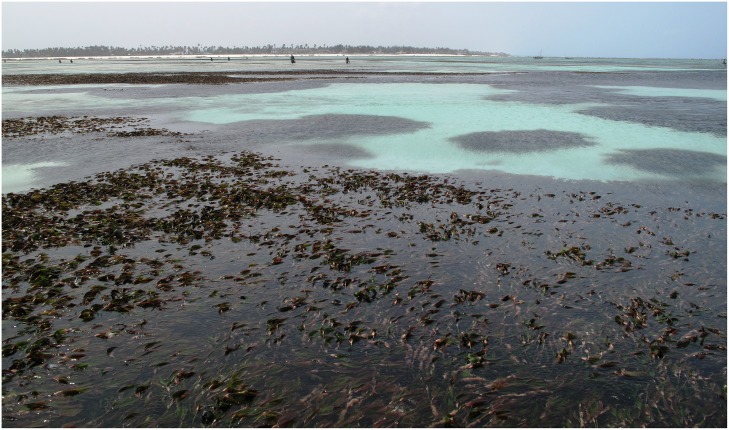
Seagrass meadow exposed during low tide. Patchy seagrass meadow dominated by *Thalassodendron ciliatum* during low tide in Zanzibar, Tanzania. Photo credit: Lina Mtwana Nordlund.

Humans are dependent on ecosystem services (ES), so understanding which ecosystem services are provided by seagrasses and how these services are distributed in space is important. Seagrasses are marine flowering plants, which form extensive meadows in shallow coastal waters on all continents except Antarctica [1], [2] (Fig 1). The intertidal to shallow subtidal location of most seagrasses allows relatively easy access and multiple uses as well as exposing seagrass ecosystems to both terrestrial and marine based threats [3], [4], [5]. The many threats to seagrass are causing it to rapidly disappear globally [3], [5], [6], [7]. Still, seagrass receives less attention than other habitats (e.g. mangrove and coral reefs) and is often not considered in coastal management decisions [3], [8], [9].

Seagrasses undoubtedly provide many ecosystem services [4], [10], defined here as natural processes and components that benefit human needs, directly or indirectly [11]. However, the variability in the provision of ecosystem services by different genera of seagrasses is largely unknown. Although seagrasses are structurally similar, they vary widely in size and productivity [12]. For example, the leaves of some genera may reach just a centimetre above the sediment surface while others extend canopies several meters into the water column. Rhizomes and roots may also penetrate and modify different depths of sediments depending on the genera. These differences in the size and productivity of seagrasses can influence all key ecosystem services, especially important services such as coastal protection, nursery habitats, and sediment accretion and stabilization [13], [14], [15].

Fortunately, the number of publications about seagrass is rapidly increasing but findings are not always presented in the context of an ecosystem service, likely due to the fact that often the focus of a study is not strictly on ecosystem services. For example, a study about trophic importance of diatoms in seagrass or research on seagrass wrack as fertilizer in the coastal areas may not present their finding as an ecosystem service [16], [17]. Thus, it remains a challenge to get an overview of existing seagrass ecosystem services, and which services arise from different genera and bioregions, from the literature.

Seagrass ecosystem services, like all other ecosystem services, are difficult to value and rank as the benefits to humans are difficult to quantify. In some areas seagrass ecosystem services, such as fish and invertebrate habitat, are crucial to the lives of the local community [4], [18], while in other areas those services are valuable but their loss would not directly affect the local communities. For accurate valuation of coastal and marine ecosystems, including seagrass ecosystems, spatial and temporal variation in the provision of services as well as synergies among ecosystem functions need to be understood and evaluated [13], [19], [20]. There have been some attempts to estimate the economic value of seagrass ecosystem services, but with limited available information accurate estimates are very difficult to obtain [10], [15], [21], [19], [22]. This suggests that there is a considerable gap in the literature when it comes to determining the contribution of seagrasses worldwide in terms of the provision of ecosystem services, or benefits, to humankind.

Here we review global seagrass ecosystem services and contrast seagrass genera to demonstrate variability in the provisioning of ecosystem services and to identify important gaps in our existing knowledge. To address this we used two approaches, a workshop that elicited information from experts and a selective literature search. Based on the expert workshop, we first identify ecosystem services known to be provided by each seagrass genus in the six different seagrass bioregions [2]. With these data, we analyze frequency and variation of seagrass ecosystem services. Based on the selective literature search, we enhance the findings from the expert workshop and create an overview with example references of ecosystem services. We thereafter discuss the variation of seagrass ecosystem services and highlight potential problems with limited knowledge about these services.

## Materials and Methods

### Definitions of the Concepts

The definition used for ecosystem services is based on the standardized framework by De Groot et al. 2002 [11]; they identified 23 ecosystem functions that provide a much larger number of goods and services, hereafter called services. They define ecosystem functions as ‘the capacity of natural processes and components to provide goods and services that satisfy human needs, directly or indirectly’ [11]. The bioregions used in this study are the six seagrass bioregions according to Short et al. (2007) [2] which is the current standard used by the international seagrass research community. These six bioregions are Temperate North Atlantic (I), Tropical Atlantic (II), Mediterranean (III), Temperate North Pacific (IV), Tropical Indo-Pacific (V), and Temperate Southern Ocean (VI), and are based on assemblages of taxonomic groups of seagrasses in temperate and tropical areas and the physical separation of the world's oceans.

### Survey of Experts

Expert knowledge is used widely in the science and practice of conservation, and eliciting opinions and information from experts is commonly used to fill knowledge gaps [8], [23], [24], [25], [26]. In this study, we have followed the five step expert-elicitation approach [25]. We use the definition of an expert proposed by Krueger et al. [24], namely “an expert can be anyone with relevant and extensive or in-depth experience in relation to a topic of interest”. Based on these criteria, we define experts as managers, practitioners and researchers working with (a) questions related to the natural or social environment of seagrass, and/or (b) questions relevant to seagrass ecosystems. Our goal for selecting seagrass experts was to include a broad range of expertise from many different fields.

To gather expert knowledge we held a workshop entitled “Seagrass ecosystem services: looking back for existing knowledge and into the future for new approaches” during the 10^th^ International Seagrass Biology Workshop (ISBW), in Buzios, Brazil in 2012. The ISBW attracts participants from academic institutions, government agencies and non-government organizations with expertise in seagrass biology, ecology, management, monitoring and social aspects of seagrass research. ISBWs take place every other year with participants from all over the world. The 91 workshop participants from 25 nations constituted most of the 101 ISBW attendees (i.e. 90%), as there were no other parallel sessions. The participation in the workshop was voluntary and before starting all participants were made aware that the results would be published in a scientific journal.

**Fig 2:**
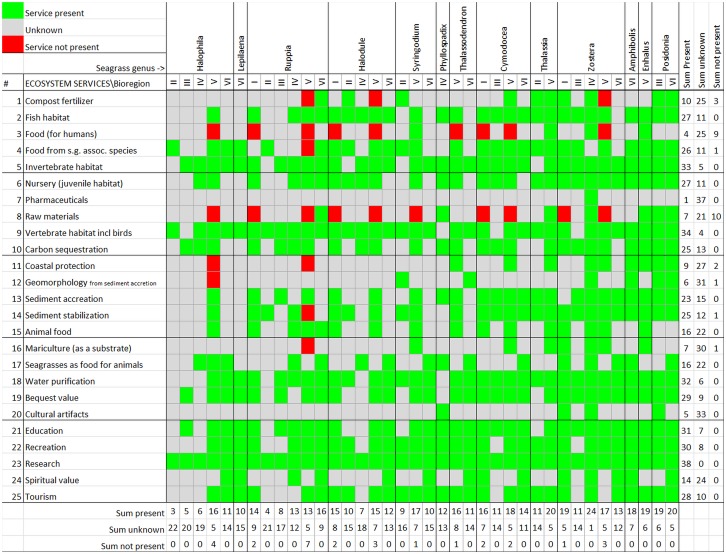
Ecosystem services (ES) provided by seagrass—expert eliciting. Colours represent consensus view of experts’ in each bioregional group. Red represents service not present; grey unknown and green service present. A sum of present, unknown, not present services scores can be seen in the table to the far right per ES and at the bottom for each genus in every bioregion. Bioregions according to Short et al., 2007 [2]:I = Temperate North Atlantic, II = Tropical Atlantic, III = Mediterranean, IV = Temperate North Pacific, V = Tropical Indo-Pacific, VI = Temperate Southern Oceans. At the far left # indicates a number that corresponds to the same ecosystem service in Table 1 facilitate comparisons, and has no prioritization.

The aim of the workshop was to survey the provision of ecosystem services by seagrass genera in different bioregions. During the first part of the workshop the goal was to identify ecosystem services known to be provided by seagrass somewhere on the planet. Through an open floor discussion, we encouraged participants to add, change or remove ecosystem services to a list provided to all participants (due to time constraints we started the workshop by presenting a preliminary list of a few ecosystem services commonly listed in the scientific literature [15], [27]). The organizers also noted when specific information about a seagrass ecosystem service was mentioned (later added to Fig 2). A final list was agreed upon by the participants. The list has no prioritization, but is arranged to have similar ecosystem services clumped. The workshop organizers then finalised an excel file that contained the list of 25 ecosystem services on the *y*-axis, and on the *x*-axis the six bioregions nested in each of the 13 seagrass genera (the skeleton of Fig 2). Even though we are aware seagrass species’ characteristics may vary (e.g. size), we focused on seagrass genera, due to time constraints, the large amount of data, paucity of knowledge of some species and to facilitate group work.

During the second part of the workshop, participants were divided into groups representing the six bioregions (and into sub-groups in highly diverse bioregions) based on their geographical working experience (i.e. where their expertise was strongest). The groups were asked to add information based on their own knowledge (through publications, ongoing research, their own research and own observations) of seagrass ecosystem service within their bioregion. They were asked to fill an excel spreadsheet and score each ecosystem service for each genus present in the bioregion. Experts gave each ecosystem service a categorical score indicating the ecosystem service was known to be present, might be present, not present, and unknown/unsure. Internet searches were allowed. Each bioregion had the following number of respondents during the second half of the workshop: seven for Temperate North Atlantic (I), eight for Tropical Atlantic (II), five for the Mediterranean (III), eight for Temperate North Pacific (IV), eleven for Tropical Indo-Pacific (V) and six for Temperate Southern Oceans (V). Results, the consensus view of all group members, were reported by the facilitators of groups from each bioregion.

We later decided to use three instead of the original four categories, namely (1) service known to be present; (2) service unknown (service might be present, ranging from unlikely to likely); (3) service known to be not present (service could not be classified even in the “unknown” category). This was done as a precaution as some experts did not distinguish between unknown and might be present. The data from all groups were then compiled into one table (Fig 2). After the workshop, the table was circulated via e-mail to an additional twelve seagrass experts that did not attend the 10th ISBW and have expertise from Africa, for potential gap filling, but the response frequency was very poor. These additional experts were also informed that their responses were to be used in a scientific publication.

The specific hypotheses in the survey of experts part of the study was: i) some ESs are more frequently present than others, independently of genera present; ii) variation exists in the provision of seagrass ESs among the globes bioregions; iii) more genera present per bioregion provide more ESs; iv) different seagrass ESs are provided by different genera and with varying frequency; v) Seagrass genus size will predict the frequency of provision of ESs.

#### Statistical analyses

The statistical analyses are based on the expert opinion data compiled in Fig 2. The original four categories were converted into three categories (see above). We used frequency of known occurrence (service present) in our analyses. Thereafter we calculated means of frequencies across the (25) services and across the (6) bioregions and/or 13 genera to empirically test the stated hypotheses (i-v). To account for unequal number of genera among bioregions, means of frequencies were also calculated relative (relativized) to the number of genera present.

Total frequencies of present, unknown and not present ES (per service) were calculated across all genera and bioregions. Total frequencies of present, unknown and not present ES for each per genera in each bioregion were calculated across all ES.

We used ANOVA and Tukey tests to compare the frequency of presence of the different perceived services (as bioregional means) and compared them with genus standardised perceived services (as bioregional means) as well as to analyse differences among means of frequencies (only presence) of the perceived relative (known) provision of different ecosystem services among bioregions. We also compared differences among means of frequencies of the perceived provision of different ecosystem services among bioregions and relativized per genus. In order to examine the multivariate relationship between frequency (only presence) of ES provision and the different genera present we carried out a Principal Components Analysis (PCA) using across bioregion genus means. To test whether seagrass genus size predicts the frequency of provision of perceived ecosystem services, we used a simple linear regression with mean leaf area as a proxy for genus size. We calculated mean leaf areas of genera from species estimates provided by Duarte [12] supplemented by data in Koch and Seeliger [28] for *Ruppia* (estimated using the formula for a cylinder surface to estimate leaf area, as it has very narrow leaves almost as wide as high).

ANOVAs and regressions were performed with SPSS Statistics v19 (IBM) and PCA using Primer 6 (Primer-E Ltd.).

### Review of Literature

Reviews are useful for the purpose of summarizing (all or parts of) available literature for a topic and can for example lead to new synthetic insights, but need to be well defined to be of high quality [29], [30], [31], [32]. After the workshop, we conducted a selective review of existing literature to enhance the expert opinion study and produce an overview of existing seagrass ecosystem services and provide examples of these services (not a comprehensive review of all available information about every service).

The specific hypotheses for this part of the study were vi) investigate which seagrass ecosystem services are present on a global scale; vii) create an overview table of seagrass ecosystem services with examples of literature for each service with an indication in which bioregion the research was done; and viii) compare whether the data obtained from the survey of experts conform to available literature and highlight additional data found in the literature search.

Two approaches were used in the literature review to reduce bias, namely searching a university database, EBSCO Discovery Service (EDS) at Stockholm University (February to June 2015) and contacting the seagrass research community for articles relevant to this study via the Seagrass Forum email-list (672 subscribers, June 2015) (March to May 2015). To define the topic and objective of the review, we identified four areas where we were to focus our search for literature. First, we wanted to investigate which seagrass ecosystem services are present on a global scale in order to be able to present exhaustive information. This was done by scrutinizing literature that might provide evidence of additional ecosystem services. The goal was to see if we had missed and to include any potential seagrass ecosystem service not found from the expert survey.

Second, we wanted to investigate existing literature to add information to ‘unknowns’ from the expert eliciting workshop. Thus, we identified areas where there were knowledge gaps or ‘service not present’ from the expert opinion survey (see Fig 2) and where we suspected underrepresentation during the workshop in terms of research field (e.g. pharmaceuticals) and geographical area (e.g. Africa). In the database search, we used the terms seagrass OR one of the 13 seagrass genera AND the service of interest. In some cases, we also used seagrass species name and country. For example, if there were gaps in the table from the workshop from bioregion X, then we tried to find scientific publications from bioregion X, as a means to collate as much information as possible. Literature identified to cover areas of knowledge gaps from the expert survey were added to Table 1 by bioregion (and not by seagrass genera) and formatted in bold typeface.

**Table 1:** Literature review of seagrass ecosystem services. The table presents a comprehensive list of ecosystem services provided by seagrass along with a selection of available research for services, each reference is followed by a parenthesis indicating the bioregion where the research was conducted. The bioregions are I = Temperate North Atlantic, II = Tropical Atlantic, III = Mediterranean, IV = Temperate North Pacific, V = Tropical Indo-Pacific, VI = Temperate Southern Oceans (Bioregions according to Short et al. 2007 [2]), R = Review of multiple bioregions. This selection of references deliberately includes only some of the references available per ecosystem service and bioregion. However, if research on this ecosystem service is common several references are included. # indicates a number that corresponds to the same ecosystem service in Figs 2 and 5. References in bold are disparities between the expert opinions and literature review, i.e. listed as unknown in the expert opinion study for a specific bioregion (it does not consider genera) or not listed as an ecosystem service in Fig 2.

#	Ecosystem service	References (a representative selection)
1	Compost fertilizer	de la Torre-Castro & Rönnbäck 2004 [33] (V); Cocozza et al 2011 [34] (III); Grassi et al 2015 [17] (III)
2	Fish habitat	Edgar & Shaw 1995 [35] (VI); Maciá & Robinson 2005 [36] (II); Ambo-Rappe et al 2013 [37] (V); Aller et al 2014 [38](V); Boström et al 2014 [39] (I); Cullen-Unsworth et al 2014 [4] (R); Jackson et al 2015 [40] (III)
3	Food (seagrass as food for humans)	**Prendergast 2002** [41] **(I** & IV); Bandeira & Gell 2003 [42] (V); Ochieng & Erftemeijer 2003 [43] (V); pers. comm. Tony Larkum—*Posidonia australis* seeds are said to have been eaten by aborigines (VI)
4	Human food from seagrass associated species (e.g. rabbitfish)	Fredriksen et al 2004 [44] (I); Nordlund et al 2010 [18] (V); Antón et al 2011 [45] (II); Lebreton et al 2012 [46] (I); Nordlund & Gullström 2013 [47] (V); Cullen-Unsworth et al 2014 [4] (R); Jackson et al 2015 [40] (III); Giakoumi et al 2015 [48] (III)
5	Invertebrate habitat	Edgar & Shaw 1995 [35] (VI); Fredriksen et al 2004 [44] (I); Boström et al 2006 [49] (I); Lavesque et al 2009 [50] (I); Nordlund et al 2010 [18] (V); Antón et al 2011 [45] (II); Albano & Sabelli 2012 [51] (III); Tu Do et al 2012 [52] (I); Gullström et al 2011 [53] (I); Lebreton et al 2012 [46] (I); Nordlund & Gullström 2013 [47] (V); Boström et al 2014 [39] (I); Cullen-Unsworth et al 2014 [4] (R); Michel et al 2014 [54] (III)
6	Nursery (habitat for juveniles)	Nakamura & Sano 2004 [55] (IV); Antón et al 2011 [45] (II); Ambo-Rappe et al 2013 [37] (V); Boström et al 2014 [39] (I); Jackson et al 2015 [40] (III)
7	Pharmaceuticals	**de la Torre-Castro & Rönnbäck 2004** [33] **(V); Kenworthy et al 2006** [56] **(R); Qi et al 2008** [57] **(V; Enhalus); Yuvaraj et al 2012** [58] **(V; Halophilia); Kannan et al 2013** [59] **(V; Halodule)**
8	Raw material	**Wyllie-Echeverria & Cox 1999** [60] **(I); Kenworthy et al 2006** [56] **(R)**
9	Vertebrate habitat incl birds (other than fish)	Bjorndal 1980 [61] (II); Dos Santos et al 2012 [62] (VI); Frazier et al 2014 [63] (IV); Christianen et al 2014 [64] (V)
10	Carbon sequestration (capturing CO2 and stores it, so called carbon sink)	Champenois & Borges 2012 [65] (III); Fourqurean et al 2012 [66] (R); Luisetti et al 2013 [67] (I, III); Gustafsson & Boström 2013 [68] (I); Boström et al 2014 [39] (I); Lutz & Martin 2014 [69] (R); Macreadie et al 2014 [70] (VI)
11	Coastal protection (e.g. wave dampening)	**Lavesque et al 2009** [50] **(I); Antón et al 2011** [45] **(II)**; Barbier et al 2011 [15] (R); **Paul & Amos 2011** [71] **(I); Tu Do et al 2012** [52] **(I)**; Christianen et al 2013 [72] (V)
12	Geomorphology as a result of sediment accretion	**Hemminga & Nieuwenhuize 1990** [73] **(II)**; Mateo et al 2003 [74] (III)
13	Sediment accretion (adding of sediment)	Van Keulen & Borowitzka 2003 [75] (VI); Barry et al 2013 [76] (II)
14	Sediment stabilization	Van Keulen & Borowitzka 2003 [75] [74] [73] (VI); Newell & Koch 2004 [77] (I); Christianen et al 2013 [72] (V)
15	Animal food from s.g. associated species	Orth et al 1984 [78] (R); Boström et al 2006 [49] (I); Lebreton et al 2011 [46] (I)
16	Mariculture (as a habitat/substrate)	de la Torre-Castro & Rönnbäck 2004 [33] (V); Eklöf et al 2006 [79] (V); Wagner et al 2012 [80] (IV)
17	Seagrasses as food for animals (e.g. dugong eats seagrass)	Bjorndal 1980 [61] (II); Thayer et al 1984 [81] (R); **Moran & Bjorndal 2007** [82] **(II)**; Martin et al 2010 [83] (I); Lebreton et al 2011 [16] (I); Lebreton et al 2012 [46] (I); Christianen et al 2014 [64] (V); Michel et al 2014 [54] (III); Giakoumi et al 2015 [48] (III)
18	Water purification	Newell & Koch 2004 [77] (I); Fernandes et al 2009 [84] (VI); Antón et al 2011 [45] (II); Richir et al 2013 [85] (III)
**new**	**Primary production**	**Gustafsson & Boström 2013** [68] **(I); Buapet et al 2013** [86] **(I)**
19	Bequest value (satisfaction of preserving seagrass)	Wyllie-Echeverria et al 1999 [87] (R); de la Torre-Castro & Rönnbäck 2004 [33] (V); Kenworthy et al 2006 [56] (R)
20	Cultural artefacts	**de la Torre-Castro & Rönnbäck 2004** [33] **(V)**
21	Education	Patterson et al 2009 [88] (V); Unsworth & Cullen 2010 [89] (V); El Shaffai 2011 [90] (V); Nordlund et al 2013 [91] (V); pers. comm. Richard Unsworth field trips with students to seagrass (I)
22	Recreation	de la Torre-Castro & Rönnbäck 2004 [33] (V); Unsworth & Cullen 2011 [92] (V); Nordlund et al 2013 [91] (V); Unsworth et al 2013 [93] (I)
23	Research	Gobert et al 2002 [94] (III); Virnstein et al 2009 [95] (II); Knudby & Nordlund 2011 [96] (V); Kaewsrikhaw & Prathep 2014 [97] (V); Nordlund et al 2014 [3] (V); Giakoumi et al 2015 [48] (III)
24	Spiritual & religious value	de la Torre-Castro & Rönnbäck 2004 [33] (V); Kenworthy et al 2006 [56] (R)
25	Tourism	Barbier et al 2011 [15] (R); El Shaffai 2011 [90] (V); Nordlund et al 2013 [91] (V); Unsworth et al 2013 [93] (I); Cullen-Unsworth et al 2014 [4] (R)
**new**	**Source of information (e.g. navigation; water quality indicator; biological sentinels)**	**de la Torre-Castro & Rönnbäck 2004** [33]**(V); Orth et al 2006** [98] **(R); Richir et al 2013** [85] **(III); Govers et al 2014** [99] **(R); Richir et al 2015** [100] **(III)**
**new**	**Genetic resources**	**Sinclair et al 2014** [101] **(VI)**

Third, we wanted to use the literature search to find and include examples of each ecosystem service identified by the experts at ISBW and as well as for additional services identified in the literature search. These examples are presented, by bioregion, in Table 1. We decided to limit the number of references to 75. We used the search terms seagrass (OR if needed one of the 13 seagrass genera, thereafter seagrass species if nothing was found for genera name) AND each of the seagrass services (if needed alternative term or word for a service). We aimed to include as many different authors as possible as examples of available literature. For example, if one author (first author) had ten papers for ten different ecosystem services then we chose to include this author only once or twice (where possible) in Table 1 and continued to search for other publications for the other services.

Fourth, we wanted to see if the data obtained from the survey of experts conform to available literature because expert knowledge may be subject to biases and errors. Searches of ecosystem services included in the list from the workshop (presented in Fig 2) were made to investigate if there were published literature about those services and provide references showing that they exist.

There was no restriction of publication date for publications to be included. There are overlaps between the experts that attended the workshop and authors of the papers included in this study. Due to space and time limitations genera was not considered, and not all bioregions are covered for each ecosystem service (as there are 950 possible combinations for all combinations of ecosystem services, genera and bioregions).

All literature received from email-list members was read and if appropriate (i.e. proving and describing a service and for a bioregion that is not heavily represented) added to this study; on some occasions we searched for other papers by that author and used those findings instead. We made an effort to include at least one paper from each of the persons that sent us citations if they met the focus areas, without regard to whether they attended the workshop or not. Every contributor included citations where they were one of the authors. About 30 out of 150 received citations were included in Table 1, as several email-list members provided several citations containing the same service from the same bioregion.

## Results

### Seagrass Ecosystem Services from Expert Workshop

The 91 experts identified 25 seagrass ecosystem services globally. The full list of services is presented in Fig 2, and contains both well-known services such as invertebrate habitat as well as largely unknown services such as the use of seagrasses for pharmaceuticals. Scores, i.e. how many times the experts consensus was: present, unknown and not present, for each of the 25 seagrass ecosystem services at genera and bioregional level are shown in Fig 2. Short explanations of some of the ecosystem services are included in Table 1. The global distribution of the 13 currently-recognized seagrass genera outlined by the experts in this study (Fig 2) are in accordance with previous research, see for example Short et al 2011 [6] and Short et al 2007 [2].

#### Some ESs are more frequently present than others, independently of genera present

The only seagrass ecosystem service that was scored to be present across all seagrass genera in all bioregions was provision for research (Fig 2) closely followed by provision of vertebrate and invertebrate habitat, water purification, education and recreation. There are several ecosystem services listed as unknown (ranging from probable to unlikely); provision of pharmaceuticals was scored as unknown for all bioregions and seagrass genera except for the genus *Zostera* in bioregion Temperate North Pacific (IV). Other largely unknown ecosystem services were the provision of cultural artefacts, geomorphology as a result of sediment accretion, mariculture, coastal protection and compost fertilizer. The experts felt strongly that seagrasses were not often used as a raw material (10 out of 38 entries scored as not present, and 21 as unknown) or food for humans (9 out of 38 not present, and 25 unknown) (Fig 2). The average number of knowledge gaps, i.e. ‘unknowns’, for all bioregions combined per genera is greater for *Halophila* and *Lepilaena*, while *Posidonia* and *Enhalus* have the least (Fig 2). The average number of knowledge gaps, i.e. ‘unknowns’, for all genera combined per bioregion is greater for bioregion Tropical Atlantic (II) and Mediterranean (III), while bioregion Temperate North Atlantic (I) and Tropical Indo-Pacific (V) have the least knowledge gaps (Fig 2).

**Fig 3:**
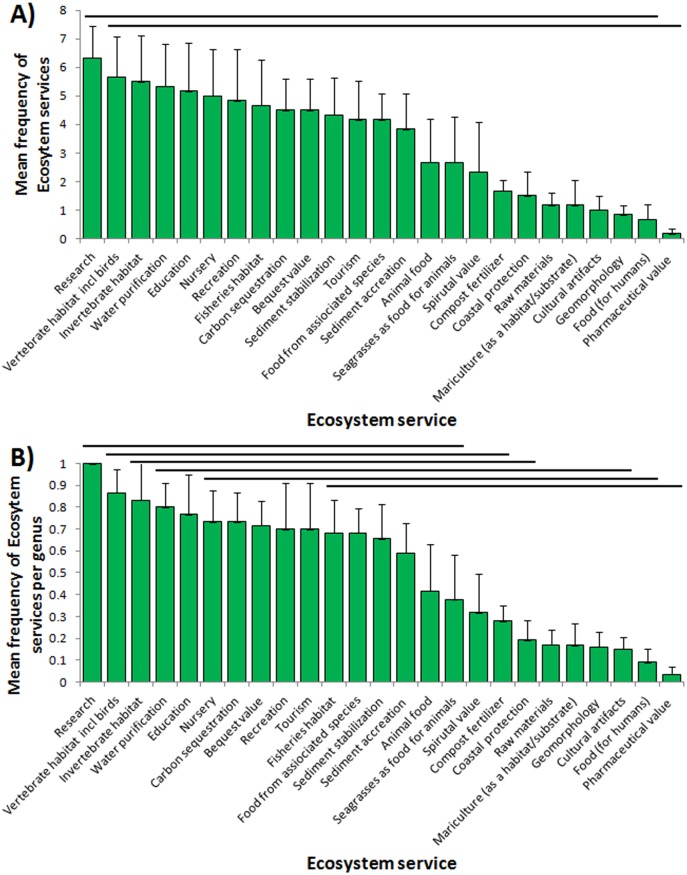
Perceived provision of seagrass ecosystem services. Global A) mean frequency, B) mean frequency per genus, of perceived provision of different ecosystem services of seagrasses. The higher mean the more frequently that service is provided. Data are across bioregion and genera means ± SE. Horizontal bars represent homogenous subsets (Tukey test).

The frequency of the global perceived relative (known) provision of different ecosystem services, overall bioregions and genus means, is presented ranked in Fig 3. This ranking provides a different view of the global value of the most and least likelihood of provisioning of ecosystem services and also their relative perceived variability. Globally, the mean frequency of provision of service differed significantly among services (ANOVA F_(24,125)_ = 2.78, p<0.001; Fig 3A) as did the mean frequency of provision of service per genus (ANOVA F_(24,125)_ = 5.40, p<0.001; Fig 3B). Relativization did not greatly influence the ranking in frequency of ecosystem services but was important for separating homogenous subsets and significant differences (compare rank order and horizontal bars Fig 3A and 3B).

#### Variation exists in the provision of seagrass ESs among global bioregions

**Fig 4:**
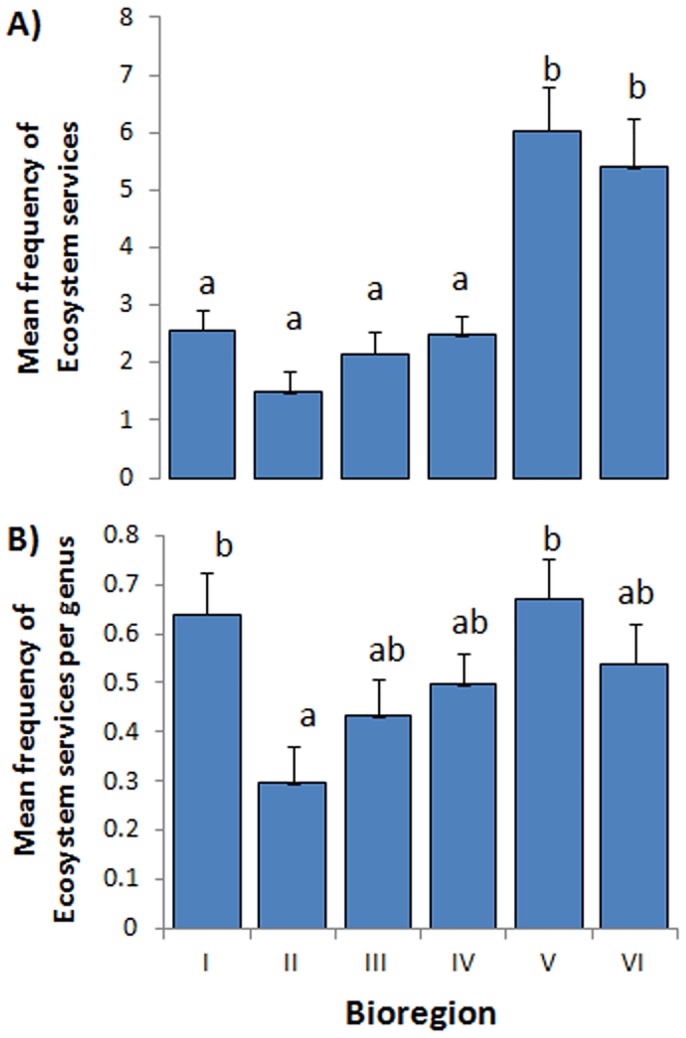
Perceived provision of seagrass ecosystem services among bioregions. A) mean frequency, B) mean frequency per genus, of perceived provision of different ecosystem services of seagrasses. Large values of mean frequency of ES show that more services are provided. Data are across service and genera means ± SE. Bars with different letters (a and b) are significantly different (Tukey test).

The mean frequency of the perceived relative (known) provision of different ecosystem services varied significantly across bioregions (ANOVA F_(5, 144)_ = 12,50, p<0.001). Specifically, seagrass in bioregions Tropical Indo-Pacific (V) and Temperate Southern Oceans (VI) was perceived to have higher levels of ecosystem services than bioregions Temperate North Atlantic (I), Tropical Atlantic (II), Mediterranean (III) and Temperate North Pacific (IV) (Tukey test; Fig 4A).

#### More genera present per bioregion provide more ESs

When the mean frequency of the perceived relative (known) provision of different ecosystem services was relativized per genus, bioregions Temperate Southern Oceans (VI) and Tropical Indo-Pacific (V) were perceived to have a higher mean frequency of seagrass ecosystem services than the bioregion Tropical Atlantic (II) (ANOVA F_(5, 144)_ = 3,20, p = 0.009; Tukey test; Fig 4B) so perceived bioregional differences were in part dependent on the number of genera present.

#### Different seagrass ESs are provided by different genera and with varying frequency

**Fig 5:**
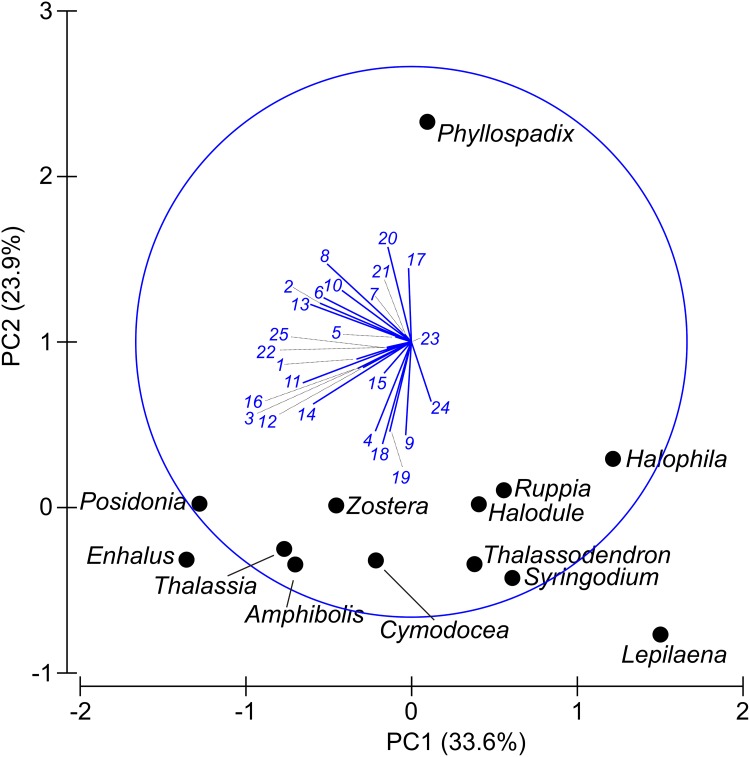
Multivariate relationship among genera and perceived seagrass ecosystem services. The following numbers correspond to the numbers in Fig 2 and Table 1. Ecosystem service: 1 = Compost fertilizer; 2 = Fisheries habitat; 3 = Food (for humans); 4 = Food from seagrass associated species; 5 = Invertebrate habitat; 6 = Nursery; 7 = Pharmaceuticals; 8 = Raw materials; 9 = Vertebrate habitat incl birds; 10 = Carbon sequestration; 11 = Coastal protection; 12 = Geomorphology as a result of sediment accretion; 13 = Sediment accretion; 14 = Sediment stabilization; 15 = Animal food; 16 = Mariculture (as a habitat/substrate); 17 = Seagrasses as food for animals; 18 = Water purification; 19 = Bequest value; 20 = Cultural artefacts; 21 = Education; 22 = Recreation; 23 = Research; 24 = Spiritual value; 25 = Tourism.

The analysis of the multivariate relationship between frequency of ES provision and the different genera present showed a clear separation (PCA eigenvalues: PC1 0.797, PC2 0.567; cumulative variation: PC1 33.6%, PC2 57.5%) among seagrass genera with a general pattern of small (right) to large (left) genera along PC1 (Fig 4). The reason for this separation is explained by the biplots (the lines originating from the centre), and there is one biplot for each ecosystem service. The biplots are mostly pointing left towards larger seagrass genera, showing that larger seagrasses such *Posidonia* and *Enhalus* are associated with the majority of ecosystem services. Larger seagrasses are especially associated with the following services: fisheries habitat (service 2), nursery (service 6), raw materials (service 8), coastal protection (service 11), sediment accretion (service 13) and sediment stabilization (service 14). *Phyllospadix* again separated from the other seagrass genera, on PCA axis 2 (PC2), mainly because compared to other seagrasses it has higher frequency of perceived cultural artefacts (service 20), seagrasses as food for animals (service 17) and value as raw materials (service 8) while the other seagrass genera have higher frequency of perceived water purification (service 18), bequest value (service 19), vertebrate habitat including birds (service 9) and food from associated species (service 4). *Lepilaena* appears on the far right because of the low frequency of perceived (known) services and its small size (Fig 5).

#### Seagrass genus size will predict the frequency of provision of ESs

**Fig 6:**
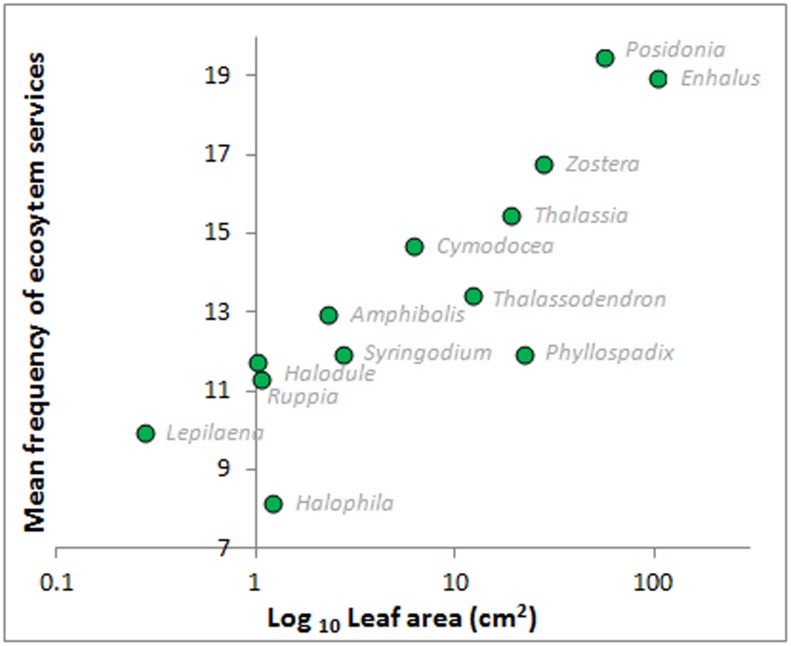
Relationship between mean perceived frequency of ecosystem service and seagrass genera shoot-specific leaf area. Relationship between mean perceived frequency of provision of ecosystem services by different seagrass genera and mean genus shoot-specific leaf area (size). Note the log10 scale (abscissa).

The analysis to test whether seagrass size explained variation in ecosystem services provisioning showed that the mean seagrass size per genus was positively associated with the mean number of known ecosystem services (linear regression: Frequency of ecosystem services = 0.0922 Leaf Area + 11.88, R² = 0.63, p = 0.001; Fig 6). The positive relation between shoot-specific total leaf area and mean ecosystem service provision suggests that *Phyllospadix* and *Halophila* were perceived to provide fewer services than would be expected by their size (as leaf area).

### Seagrass Ecosystem Services in the Literature

The literature review of seagrass ecosystem services is compiled in Table 1. Table 1 shows a comprehensive list of 28 ecosystem services provided by seagrasses. The services identified are based on the combined results of the expert workshop, literature search and the email-list request. Table 1 has a selection of papers for each ecosystem service providing examples of this ecosystem service.

During the literature search we experienced a large difference in available scientific publications among seagrass species (not only among genera) relevant to seagrass ecosystem services. For example there is a large amount of available data about *Zostera marina* (≈3000 hits in Web of Science) in comparison to several species belonging to the genera of *Lepilaena* (6 hits for genera name), *Ruppia* (≈500), *Halophila* (≈1500) and *Halodule* (≈600). The later four genera include all nine species of seagrass that are listed on the Red List of Threatened Species (all listed as data deficient) [6]. Furthermore, the amount of information in the scientific literature available for the different ecosystem services provided by seagrass also varies greatly. Scientific papers about seagrass as fish, invertebrate and vertebrate (other than fish) habitat, human food from seagrass associated species are commonly available, while there are fewer papers about seagrasses providing pharmaceuticals, geomorphology and cultural services (compare search for seagrass AND fish AND habitat ≈1400 hits vs seagrass AND pharmaceutical 10 hits in Web of Science).

We also found differences in available scientific publications on a geographical scale, commonly with less data available in remote geographical areas and from undeveloped countries (even though the large and diverse bioregion, Tropical Indo-Pacific (V), has many publications in total). Bioregion Tropical Atlantic (II), Temperate North Pacific (IV) and Temperate Southern Oceans (VI) tend to be less represented in the literature. Another insight is that the Tropical Indo-Pacific bioregion (V) has a lot more available data on cultural services, such as spiritual value, than other bioregions, which probably indicates that the people living in this bioregion depend more directly on seagrass habitat for their livelihoods.

### Expert Survey vs. Literature Review

In general the expert survey (Fig 2) conforms to the literature review (Table 1), although there is some disparity between the two. This shows that the expert opinions are reliable.

This study only presents a comparison of the expert survey to the literature review. The literature review produced three additional ecosystem services compared to the expert opinion study, namely genetic resources, primary production and source of information (e.g. navigation; water quality indicator; biological sentinels), totalling 28 services. The disparities are highlighted in bold in Table 1, thus pointing out additional information compared to the data presented in Fig 2. The expert opinion survey allowed us to include as yet unpublished data and information based on experience of the experts, information that may be currently impossible to find in the literature. This is a great advantage as most studies submitted and/or published have significant results. For example, it is more likely to study and publish the presence and details about an existing service than the absence of a seagrass ecosystem service, due to publication bias [102].

With the literature review, we could fill some of the knowledge gaps from the expert workshop, regarding pharmaceuticals, seagrass as raw material, coastal protection, and geomorphology as a result of sediment accretion. The expert survey and the literature review together present extensive information about seagrass ecosystem services.

## Discussion

The human benefits of seagrasses are extensive, and seagrasses may be even more valuable than previously thought as published papers about ecological and economic value of seagrass may not include or consider all ecosystem services present [15], [27], [103]. Comparing our findings with a conceptual framework and typology for describing, classifying and valuing ecosystem functions, goods and services in general [11] we see that seagrasses contribute to all 23 ecosystem functions except pollination. Our review and expert study shows that frequently documented ecosystem services were often those that are associated with faunas that are considered important or appealing to humans. The relatively few scores of “not present” in the expert study might reflect the low interest in researching (or the possibility to publish) non-existing services instead of existing services, as well as the deep understanding among the experts that more research might reveal presence of services that have not yet been demonstrated. Some research fields scored more unknowns by the seagrass experts such as pharmaceutical research and geomorphology (that use or depend on seagrasses). For example, molecular biologists are conducting research about extraction of pharmaceuticals from seagrasses, but these substances are not yet used in medicine, the experts seem well aware of the potential but not the fact that pharmaceuticals from some species in some bioregions have already been identified. The cultural ecosystem services might also be underrepresented in the expert survey as those services tend to be assessed mainly by social scientists, whereas many seagrass experts categorise themselves as natural scientists. Furthermore, cultural services may also be very difficult to assess and quantify [104], [105]. These findings highlight the need for more inter-disciplinary research and frameworks on how to assess and quantify such services.

The global scope of this study allows us to tease out genera- and bioregional-specific differences and our results indicate that seagrass ecosystem services, and thus the human benefits, vary across genera and geographical areas. We found that variation in plant size per seagrass genera (i.e. shoot-specific total leaf area of seagrass shoots) was positively associated with the number of (perceived) ecosystem services. However, two genera *Phyllospadix* and *Halophila* had fewer ecosystem services than expected based on the size relationship. This is likely related to genus-specific morphological or ecological characteristics. *Phyllospadix* (surfgrass) is the only seagrass that grows on rocky shores rather than in soil and has a suite of fundamentally different perceived ecosystem services (see Fig 5). Smaller sized seagrasses (total leaf area) such as *Halophila* and *Lepilaena*, have more ‘unknowns’ than larger species such *Posidonia* and *Enhalus*. This may indicate that smaller species provide fewer services, lower quality of services, and/or that past research has tended to focus on larger species. This does not mean that smaller species are less important. Importance is difficult to assess because it relates to complex differences in perceptions, cultures, and regions that are beyond the scope of this study. However, a smaller species may provide a service considered very valuable while a larger species may not. For example, the charismatic dugongs and manatees prefer to feed on smaller seagrass species such as *Halophila* [106]. Our findings clearly point out the need for more research on smaller sized seagrass species.

Geographical differences in seagrass services seem to be both a function of the genera occurring in each bioregion as well as the perception among experts of some fundamentally different provision of services or lack of them around the globe. Remote and undeveloped areas tend to have less available scientific data (even if people are living in close relation with the seagrass and may have extensive knowledge). The Tropical Atlantic (II) and Mediterranean (III) bioregions stick out in having more unknowns on average (all genera combined). *Posidonia* in the Mediterranean has far more known services than other species in that bioregion, pointing out the unbalanced research effort for genera. The reason for this might be the extensive distribution and the large plant size of *Posidonia*. These findings point to the need for more balanced research of different geographical areas, especially of low-income countries and remote areas, and different species, regardless of size and distribution.

We believe that the common understanding of the importance of seagrasses is still in its infancy. Our study points out the many knowledge gaps about seagrass ecosystem services, and this pattern would likely be even clearer if all seagrass species were included, because so many seagrass species remain understudied. The extensive global distribution of seagrasses from the coldest to the warmest ocean waters, the ability to grow from the intertidal zone down to approximately 60 meters, and the fact that seagrasses are flowering plants with roots, rhizome and leaves [107], makes detailed comprehensive studies of seagrasses challenging. Therefore, future research opportunities are plenty, not yet mentioned in this paper are the likely influence of size and density of the seagrass meadow on the provision of ecosystem services. Furthermore, intra-specific variation in ecosystem function may be important for the provision of seagrass ecosystem services. For example, *Zostera marina* colonizing the cold waters off Maine and New Hampshire (USA) have leaves that grow more than 1 m long, but *Z*. *marina* growing in the warmer waters of Maryland and North Carolina (USA) typically produce leaves < 30 cm and have very different total leaf areas [108], [109]. Furthermore, *Z*. *marina* in the Baltic Sea shed most of their leaves during the winter while in other regions they persist throughout the year. Seasonal and temporal differences (e.g. the influence of low versus high-tides) on the flow of ecosystem services would be valuable to research further, as there is some evidence that such variations influence provision of seagrass ecosystem services [7], [19]. As our results indicate that mean genus leaf area is strongly related to seagrass ecosystem services, such intra-specific variation, along with for example seasonal changes could impact the provision of ecosystem services and thus affect valuation of these benefits. Moreover, the below ground characteristics might also be highly relevant to consider, as it may have great impact on for example carbon sequestration, infauna habitat and geochemical processes [47], [72], [110].

In the literature, seagrass ecosystem services are often presented for seagrasses in general without any indication of variation in provision, such as differences among species, genera or geographical location. This may give the impression that all seagrass species provide the services mentioned, and that seagrasses provide the full range of services throughout the year, in any habitat they colonize and over all geographical regions. Our results show that all seagrasses do not provide all services, neither in all bioregions nor for all seagrass species, and that there are still substantial knowledge gaps regarding seagrass ecosystem services. We therefore suggest some caution when presenting or introducing seagrass ecosystem services to avoid such confusion. Furthermore, the geographical differences of provision of seagrass ecosystem services imply, depending on the management goals, that managers should investigate which services their seagrasses actually provide and not just rely on information on services by seagrasses in general.

## Conclusion

In summary, seagrasses produce a wide variety of ecosystem services, but not all seagrasses are equal. Larger seagrasses tend to provide a wider variety of ecosystem services than smaller ones. The provisioning of several seagrass ecosystem services appears to vary across genera and bioregions. Nevertheless, smaller seagrasses provide important ecosystem services which should be acknowledged. Our findings have the following implications for the management and economic valuation of seagrasses:

1. Gaps exist in our knowledge of the ecosystem services provided by seagrass ecosystems globally. A large proportion of the identified ecosystem services have unknown provision for some genera and bioregions Further research is required to determine whether these services are not provided by these genera and bioregions or whether our knowledge about these services is simply incomplete.
2. Better understanding of which ecosystem services are associated with specific seagrass genera and bioregions is important for improved coastal management and conservation. For example, if the management objective is to protect coastlines in the Tropical Indo-Pacific (bioregion V) then it may be ineffective to conserve *Halophila* or *Ruppia* and expect them to improve coastline stability. On the other hand, if the management objective is to preserve dugongs, it is important to conserve *Halophila*.
3. The transfer of estimates of economic value of services from one seagrass ecosystem to another system, genera and bioregion must be used with caution, as the lack of such ecological or economic correspondence can lead to highly unreliable valuation estimates [13], [20]. There are few comprehensive seagrass valuation studies. Existing studies commonly focus on or include only a few services and often seagrasses in general or a specific species, not considering genera or several species [111], [112]. Unreliable estimates imply that the public, managers and policy makers may be misled or confused which may affect their decision making processes. The considerable variation in seagrass ecosystem services across genera and bioregions demands that regional and species-specific valuation studies assess the benefits of seagrass systems and the multitude of species they contain.
