# Correction: Seagrass Ecosystem Services and Their Variability across Genera and Geographical Regions

**DOI:** 10.1371/journal.pone.0169942

**Published:** 2017-01-05

**Authors:** Lina Mtwana Nordlund, Evamaria W. Koch, Edward B. Barbier, Joel C. Creed

The first author’s name is incorrect in the citation. The first author in the citation should be Nordlund LM. The correct citation is: Nordlund LM, Koch EW, Barbier EB, Creed JC (2016) Seagrass Ecosystem Services and Their Variability across Genera and Geographical Regions. PLoS ONE 11(10): e0163091. doi:10.1371/journal.pone.0163091.
